# Dysregulated neuromodulation in the anterior cingulate cortex in chronic pain

**DOI:** 10.3389/fphar.2023.1289218

**Published:** 2023-10-25

**Authors:** Kevin Lançon, Philippe Séguéla

**Affiliations:** Department of Neurology and Neurosurgery, Alan Edwards Centre for Research on Pain, Montréal Neurological Institute, McGill University, Montréal, QC, Canada

**Keywords:** chronic pain, anterior cingulate cortex, analgesia, dopamine, norepinephrine, serotonin, acetylcholine, monoamines

## Abstract

Chronic pain is a significant global socioeconomic burden with limited long-term treatment options. The intractable nature of chronic pain stems from two primary factors: the multifaceted nature of pain itself and an insufficient understanding of the diverse physiological mechanisms that underlie its initiation and maintenance, in both the peripheral and central nervous systems. The development of novel non-opioidergic analgesic approaches is contingent on our ability to normalize the dysregulated nociceptive pathways involved in pathological pain processing. The anterior cingulate cortex (ACC) stands out due to its involvement in top-down modulation of pain perception, its abnormal activity in chronic pain conditions, and its contribution to cognitive functions frequently impaired in chronic pain states. Here, we review the roles of the monoamines dopamine (DA), norepinephrine (NE), serotonin (5-HT), and other neuromodulators in controlling the activity of the ACC and how chronic pain alters their signaling in ACC circuits to promote pathological hyperexcitability. Additionally, we discuss the potential of targeting these monoaminergic pathways as a therapeutic strategy for treating the cognitive and affective symptoms associated with chronic pain.

## Introduction

Chronic pain remains a significant global socioeconomic burden, afflicting millions of individuals worldwide, and effective long-term treatment options remain elusive (Holmes, 2016; Dahlhamer et al., 2018; Nahin et al., 2023). The complexity of chronic pain arises from its multifaceted nature, encompassing sensory and emotional components that involve distinct regions of the central and peripheral nervous systems. While there have been notable advancements in identifying neuronal pathways associated with chronic pain, the precise pathophysiological mechanisms inducing their dysfunction are diverse and not well established. As a result, the development of effective non-opioidergic pharmacological treatments to alleviate chronic pain symptoms has been hindered.

Recent technological advances have provided unprecedented opportunities for investigating the cortical networks involved in nociception, as well as those dysregulated by chronic pain. Significant progress has been made in identifying key regions of the “pain matrix,” a network of supraspinal regions involved in nociceptive signaling that participate in promoting pathological pain processing (Kuner and Flor, 2017; Kuner and Tan, 2020; Mercer et al., 2021).

Within the pain matrix, the anterior cingulate cortex (ACC), a region of the medial prefrontal cortex (mPFC), is particularly relevant due to its role in top-down pain modulation and its ability to regulate executive functions commonly disrupted in chronic pain (Rainville et al., 1997; Kummer et al., 2020; Phelps et al., 2021). Several studies have shown that, following neuropathic injury, pyramidal neurons in the ACC become hyperexcitable, and functional imaging studies have identified the ACC as one of the most constitutively active brain regions in chronic pain patients (Cordeiro Matos et al., 2015a; Jensen et al., 2016; Ferraro et al., 2021; Lançon et al., 2021).

Emerging evidence suggest a link between alterations in the cortical function of neuromodulators such as the monoamines dopamine (DA), norepinephrine (NE), serotonin (5-HT), and chronic pain-related cortical hyperexcitability (Ong et a, 2019; Kuner and Tan, 2020; Yang et al., 2020). DA and NE appear particularly important due to their molecularly antagonistic, yet functionally synergistic role in modulating pyramidal activity in the ACC. These neuromodulators and others, such as acetylcholine (ACh) and the neuropeptide oxytocin, are crucial in adjusting the activity of ACC circuits by balancing excitatory and inhibitory inputs on pyramidal neurons and thus play a major role in tuning pain perception. Additionally, chronic pain is linked to a high prevalence of cognitive disabilities also observed in other disorders that impact the release of neuromodulators (Hart et al., 2003; Cools and Arnsten, 2022). Affect, attention, memory, executive planning, spatial awareness, sensory discrimination, and other cognitive functions heavily influenced by cortical monoamines are common issues in chronic pain patients (Jarcho et al., 2012; Bushnell et al., 2013; Markovic et al., 2021). Our current treatments for chronic pain can be viable in the short term but lose effectiveness and create tolerance and addiction in the long term (Varrassi et al., 2010; Speed et al., 2018). Furthermore, current pain treatments do not effectively alleviate the cognitive deficits associated with chronic pain (Chapman et al., 2002; Schiltenwolf et al., 2014).

Here we discuss the involvement of the three major monoamines (DA, NE, and 5-HT), as well as ACh and oxytocin, in promoting hyperexcitability in the ACC, leading to pathological pain processing and cognitive deficits in chronic pain conditions. We will review the complex interplay between neuromodulation of ACC circuits and pain processing, ultimately fostering better understanding of the cortical causes of chronic pain at the cellular and molecular level, to aid the development of novel and more effective interventions for chronic pain management.

## Dysregulated ACC major contributor to pathological pain processing

The mPFC, and especially the ACC, is a critical hub for attention, emotional regulation, and pain modulation (Rainville et al., 1997; Stevens et al., 2011; Arnsten and Katya, 2012). Neuroimaging studies consistently report ACC activation following pain stimulation (Casey, 1999; Bastuji et al., 2016). Afferents from the medial thalamus and the somatosensory cortex relay noxious signals to the ACC, which then filters them and relays them back down to other cortical or subcortical regions involved in pain perception. These include, but are not limited to, the periaqueductal grey (PAG), the midbrain, the parabrachial nucleus (PBN), the dorsal horn of the spinal cord (DHSC), and other cortical areas such as the prelimbic, somatosensory, and infralimbic cortices (Kuner et al., 2020).

While the ACC predominantly mediates the affective dimension of nociception, it has also been shown to modulate its sensory component (Rainville et al., 1997; Chen et al., 2018; Singh et al., 2020). If the output of the ACC becomes dysregulated, the downstream signals processed by its target regions will be disrupted and will promote abnormal nociception. For example, experimental *in vivo* stimulation of pyramidal neurons in the ACC through opto- and chemogenetic actuators consistently leads to a decrease in mechanical withdrawal thresholds as well as the induction of conditioned place aversion (CPA), established readouts for the sensory and affective aspects of pain, respectively (Johansen et al., 2001; Kang et al., 2015). The ACC is pathologically hyperactive in the case of patients with nerve injuries or other chronic illnesses known to cause prolonged painful states (Jensen et al., 2016; Tan and Kuner, 2021). Decreasing this hyperactivity in animal models of chronic pain reduces both allodynia (abnormal sensitivity to innocuous stimuli) and hyperalgesia (increased sensitivity to noxious stimuli), thereby demonstrating that dysregulated ACC circuits contribute to abnormal pain processing (Kang et al., 2015; Sellmeiher et al., 2018; Juarez-Salinas et al., 2019).

This increase in excitability is caused by changes in the balance of excitatory and inhibitory inputs (E/I balance) as well as intrinsic changes in the excitability of pyramidal neurons. Following nerve injury, the E/I balance in the ACC is shifted due to enhanced excitatory glutamate release and decreased inhibitory gamma-aminobutyric acid (GABA) release. The increase in glutamate release induces a robust facilitation of long-term potentiation (LTP) that is correlated with increases in NMDA-R currents and an upregulation of the GluN2B subunit (Kuner and Flor, 2017; Zhuo, 2020; Chen et al., 2021). For GABA release, although neuropathic pain does not appear to decrease the excitability of GABAergic interneurons, there is evidence of disinhibition due to loss of GABAergic synapses (Blom et al., 2014). The intrinsic excitability of synaptically isolated pyramidal neurons in the ACC is also potentiated following nerve injury. Hyperpolarization-activated cyclic nucleotide-gated (HCN) channels, dendritic cation channels known to tune the excitability of neurons by controlling their input resistance, are dysfunctional in neuropathic states (Cordeiro-Matos et al., 2015b; Santello and Nevian, 2015; Santello et al., 2017; Lançon et al., 2021). Since HCN channel activity is controlled by the ratio of G_s_ to G_i_-linked metabotropic signaling, up and down regulators of adenyl cyclase respectively, this strongly suggests that G protein-coupled receptor activation is altered in the ACC in neuropathic states.

Identifying the factors that promote abnormal GPCR signaling in chronic pain conditions could be key to the development of novel analgesics or treatments that impede the instigation or maintenance of the pathological phenotype following nerve injury.

## Dopamine

**FIGURE 1:**
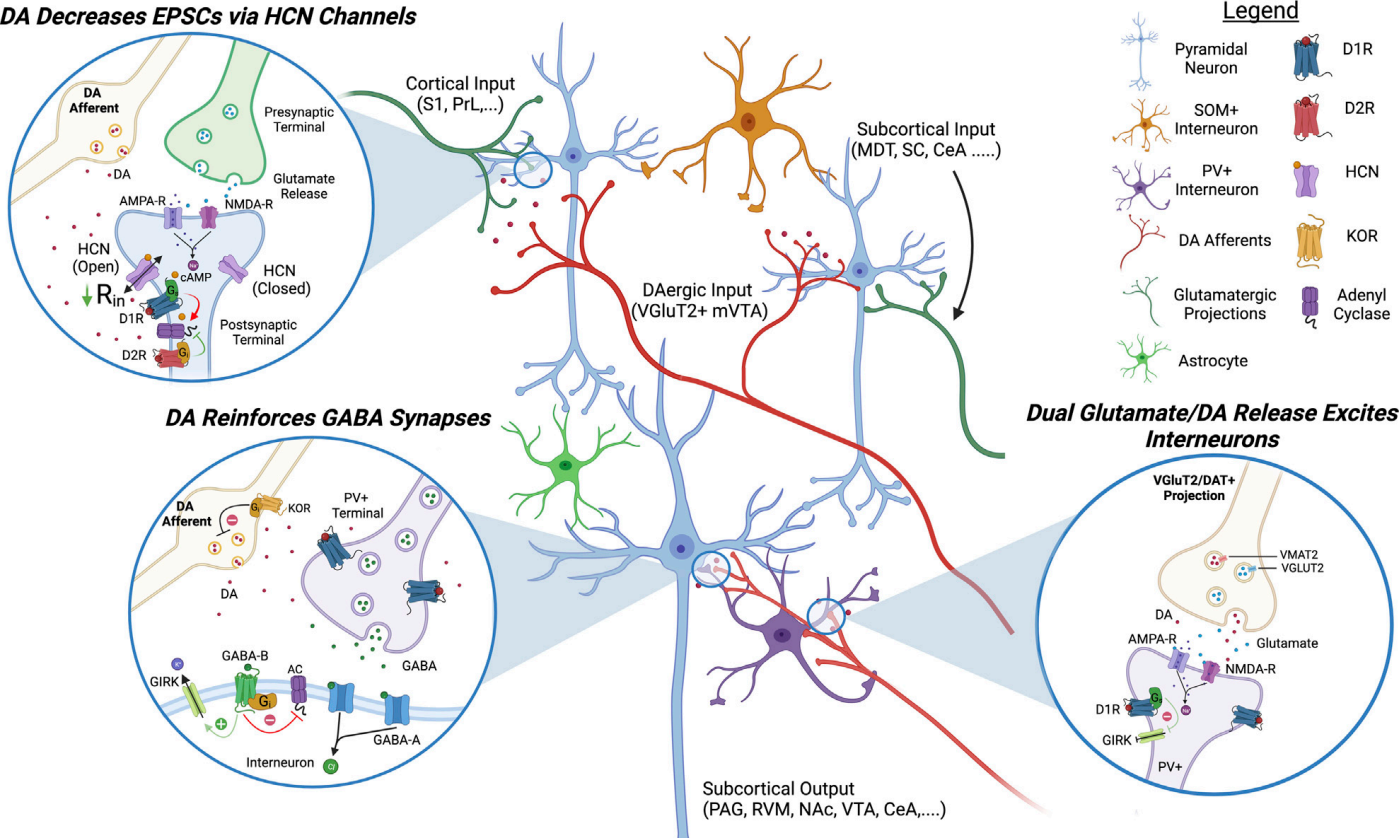
**Proposed Model of DAergic Modulation of ACC Circuits**. DAergic signaling in the ACC inhibits pyramidal neurons and activates inhibitory interneurons. D1R activation decreases EPSCs on pyramidal neurons by increasing the open channel probability of HCN channels and activates GABAergic interneurons via glutamatergic signaling and inhibition of K+ channels. DA, dopamine; HCN, hyperpolarization-activated cyclic nucleotide-gated channels; AC, adenyl cyclase; GIRK, G-protein coupled inward rectifying potassium channels; KOR, kappa opioid receptor; PAG, periaqueductal gray; S1, somatosensory cortex 1; PrL, prelimbic cortex; MDT, medial dorsal thalamus; SC, spinal cord; CeA, central nucleus of amygdala; RVM, rostral ventromedial medulla; NAc, nucleus accumbens; VTA, ventral tegmental area.

The role of DA in modulating mPFC function is relatively well studied in cognitive and attentional disorders, and more recently, in pain (Figure 1) (Vijayraghavan et al., 2007; Huang et al., 2019a). A genetically defined subset of VGluT2/DAT+ projection neurons located in the medial ventral tegmental area (VTA) has been reported to innervate the ACC and this mesocortical pathway is hypothesized to underlie salience and tune attention (Horvitz, 2000; Arnsten and Pliszka, 2011; Poulin et al., 2018). This pathway is now being studied further in its response to pain states and whether it is dysfunctional in chronic pain conditions. A recent study indicates that acute pain induces an inhibition of VTA neurons indirectly via the PBN and the substantia nigra (SNr) (Yang et al., 2021). In line with this, the VTA is hypoactive in chronic pain conditions and HPLC analysis demonstrates that DA levels are reduced in the ACC 3 days following CFA injection to evoke inflammatory pain (Narita et al., 2003; Ko et al., 2018; Huang et al., 2019b; Darvish-Ghane et al., 2020). Human imaging studies have also indicated a reduced activity of the VTA following salient stimulation in multiple forms of chronic pain (Loggia et al., 2014; Martikainen et al., 2015). Given these findings, it becomes critical to understand the role of DA in modulating ACC circuits and the impact that decreased cortical DAergic signaling would have on cortical output in chronic pain states.

G_s_-coupled DA receptor subtype 1 (D1R) is the most studied DA receptor in the ACC and is expressed on pyramidal neurons in layers 2/3 and L5/6 as well as GABAergic interneurons (Muly et al., 1998; Clarkson et al., 2017). In pyramidal neurons, D1Rs are co-localized with HCN channels on dendritic spines and electrophysiological studies have demonstrated that their activation increases the open channel probability of HCN channels, decreasing the input resistance (Paspalas et al., 2012; Lançon et al., 2021). Functionally, activation of D1R expressed on pyramidal ACC inhibits excitatory post synaptic currents (EPSCs), AMPA-R currents, and causes general inhibition by increasing the rheobase of pyramidal neurons (Darvish-Ghane et al., 2016; Lançon et al., 2021). D1Rs are also expressed on parvalbumin (PV+) fast-spiking GABAergic interneurons in the ACC (Le Moine and Gaspar, 1998; Glausier et al., 2009; Santana et al., 2009). Activation of D1Rs expressed on GABAergic interneurons inhibits leak K+ channels and inward-rectifying K+ channels to induce a robust excitation and increase inhibitory post synaptic currents (IPSCs) on pyramidal neurons (Seamans et al., 2001; Gorelova et al., 2002; Kröner et al., 2007; Ren et al., 2018; Satoh et al., 2018). In chronic pain conditions, overall D1R activation in the ACC appears to play a critical role in controlling pain perception and pain relief. Not only is microinjection of D1R agonists in the ACC analgesic but D1R signaling in the ACC is required for effective pain relief in neuropathic mice (Lançon et al., 2021). Conversely, conditional KO of D1Rs in the ACC using a crisper/cas9 construct is hyperalgesic (Darvish-Ghane et al., 2020).

G_i_-coupled DA receptor subtype 2 (D2Rs) is also expressed on PV+ interneurons but its expression decreases with age and is thought to play a role in the development of cortical circuits and in cognitive disorders such as schizophrenia in adulthood (Le Moine and Gaspar, 1998; Porter et al., 1999; Santana et al., 2009; Graham et al., 2015). Activation of D2Rs on interneurons has been shown to decrease IPSCs on pyramidal neurons (Seamans et al., 2001). D2Rs are also expressed on pyramidal neurons and their activation appears to play an excitatory role but the effect appears less robust than D1R activation (Green et al., 2020; Lançon et al., 2021). DA receptor subtype 3 (D3Rs) are also present in L2/3 ACC and although less is known about their direct effect on neuron excitability, they appear to play a role in gating the release of DA, NE, and ACh (Lacroix et al., 2003). The DA receptor subtype 5 (D5R) is expressed on pyramidal and might play a similar role as the D1R (Sarinana and Tonegawa, 2016).

In chronic pain D1R mRNA is decreased whereas D2R mRNA is increased in the ACC (Ortega-Legaspi et al., 2011). In line with this, D1R-mediated inhibition of AMPA-R currents is reduced in inflammatory pain models and other evidence indicates that D1R-evoked IPSCs are reduced in high-stress models (Satoh et al., 2018; Darvish-Ghane et al., 2022). All data converge to conclude that chronic pain is inducing a hypodopaminergic state in the ACC. Given that D2Rs have a higher sensitivity for DA than D1Rs, this hypodopaminergic state likely leads to a decrease in G_s_-coupled signaling and an increase in G_i_-coupled signaling. Activating DAergic projections in the mPFC, or activating DA neurons in the VTA directly, is analgesic in chronic pain models, thereby demonstrating that increasing cortical DA signaling is sufficient to occlude chronic pain symptoms (López-Avila et al., 2004; Huang et al., 2020).

What is causing a hypoactive VTA in chronic pain remains unclear but could be due to recruitment of the endogenous opioid system, with both the kappa opioid receptors (KORs) and the mu opioid receptors (MORs) playing a role. Dynorphin, the endogenous agonist for the G_i_-coupled KOR, is well known for its role in gating DA release (Xi et al., 1998). The KOR is highly expressed on DA terminals within the mesolimbic pathway where its activation inhibits the release of DA. In line with this, selective KOR agonists have been shown to be highly aversive in both humans and rodents and this aversion is dependent on KOR expression in DA neurons (Liu et al., 2019). In the ACC, prodynorphin mRNA is increased following nerve injury, suggesting KOR over-activation could play a role in decreasing mesocortical DA release in chronic pain conditions (Palmisano et al., 2019). Actually, a recent study demonstrates that microinjection of a KOR antagonist in the ACC is analgesic in neuropathic mice while microinjection of a KOR agonist can produce a neuropathic-like phenotype in naïve mice (Navratilova et al., 2023). MORs, other G_i_-coupled opioidergic receptors, are expressed on GABAergic neurons in the VTA and their activation inhibits GABA release, causing disinhibition of DAergic projections (Johnson and North, 1992; Narita et al., 2001). In chronic pain, MOR activation is decreased in the VTA, possibly mediated by MOR internalization, causing an elevation of synaptic GABA levels (Ozaki et al., 2002; Zuo, 2005). It is also possible that increased activity of the locus coeruleus (LC), the main source of central NE, is contributing to a hypoactive VTA in chronic pain. There is evidence of LC projections to the VTA and NE application on VTA neurons decreases the firing activity of DA neurons (Guiard et al., 2008).

Interestingly, there is a high comorbidity between several hypodopaminergic disorders, including Parkinson’s disease and major depressive disorder, and chronic pain (Miller and Cano, 2009; Ford, 2010). Parkinsonian patients have an exceptionally high incidence of non-myogenic chronic pain and fMRI studies demonstrate their ACC is hyperactive (Schestatsky et al., 2007; Blanchet and Brefel-Courbon, 2018). Supplementing supraspinal DA with L-DOPA decreases the sensory symptoms of chronic pain alongside ACC activity in Parkinsonian patients (Brefel-Courbon et al., 2005). In line with this, supplementing neuropathic mice with L-DOPA decreases chronic pain symptoms and ACC excitability (Lançon et al., 2021). As a proof of concept, it has also recently been shown that low doses of L-DOPA can reduce symptoms of chronic back pain in humans as well (Reckziegal et al., 2019). However, due to the known effects of long-term L-DOPA treatment on fine motor control, a more restrained approach to increasing cortical DA will be needed to reduce side effects.

## Norepinephrine

**FIGURE 2:**
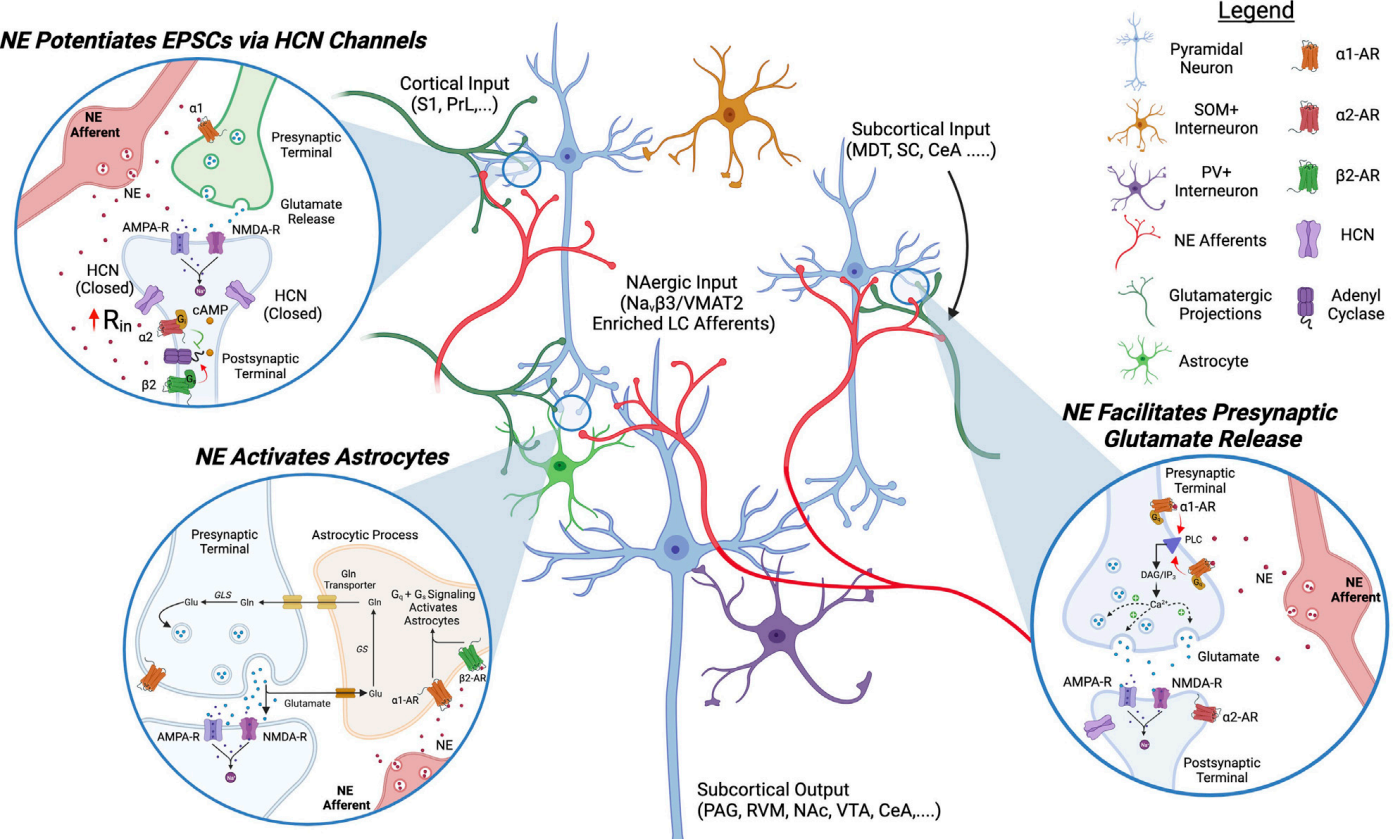
**Proposed Model of NAergic Modulation of ACC Circuits**. NAergic signaling in the ACC excites pyramidal neurons and activates astrocytes. α2 and α1 receptor activation increases EPSCs on pyramidal neurons by decreasing the open channel probability of HCN channels and promoting presynaptic glutamate release, respectively. α1 and β2 signaling activate astrocytes. NE, norepinephrine; HCN, hyperpolarization-activated cyclic nucleotide-gated channels; AC, adenyl cyclase; Gln, glutamine; PLC, phospholipase C; PAG, periaqueductal gray; S1, somatosensory cortex 1; PrL, prelimbic cortex; MDT, medial dorsal thalamus; SC, spinal cord; CeA, central nucleus of amygdala; RVM, rostral ventromedial medulla; NAc, nucleus accumbens; VTA, ventral tegmental area.

NE is another monoamine with a robust effect on supraspinal pain circuits and it appears dysregulated in chronic pain conditions (Figure 2). Similar to the VTA, the LC has high cellular heterogeneity with projection-specific neuronal subsets. Whereas spinal-projecting LC neurons are antinociceptive (see the analgesic effects of α2 agonist clonidine injected intrathecally), cortically-projecting LC neurons are pro-nociceptive (Filos et al., 1992; De Kock et al., 2005; Hirschberg et al., 2017; Koga et al., 2020). Although the LC has a less defined topographic organization than the VTA, transcriptomic studies have shown that LC projections to the mPFC express high levels of both VMAT2 and the sodium channel accessory subunit Na_v_β3, allowing them to fire at high frequencies for prolonged periods (Kole et al., 2008; Chandler, 2016). In chronic pain, the LC displays increased excitability and seems to play a role in pathological pain processing as stimulation of LC projections to the ACC increases glutamatergic transmission and induces sensitization to pain and itch (Hirschberg et al., 2017; Koga et al., 2020; Camarena-Delgado et al., 2022; Iqbal et al., 2023).

The predominant adrenergic receptors in the ACC are the GPCRs β2, α1, and α2. The G_q_-coupled α1 receptor is expressed in L2/3/5/6 on glutamatergic terminals where its activation promotes the release of glutamate, increasing EPSCs (Santana et al., 2013; Zhang et al., 2013). The G_i_-coupled α2 receptor is found on pyramidal neurons in L2/3/5/6 of the ACC and its activation has the opposite effect of D1R on HCN channels, i.e., closing them (Wang et al., 2007). α2 agonists inhibit HCN channels, increase input resistance, and increase the excitability of pyramidal neurons in the ACC (Zhang et al., 2013). The synergistic role of α1 and α2 activation leads NE to have a robust excitatory drive on ACC pyramidal and consequently on pain perception (Zhang et al., 2013; Kaushal et al., 2016).

Astrocytes and microglia play a key role in synapse reinforcement and pruning in cortical circuits and these non-neuronal cell types are also modulated by NE in the ACC and have been shown to play a role in tuning pain-induced aversion (Romanos et al., 2020; Iqbal et al., 2023). β2 and α1 receptors are expressed on astrocytes and their activation induces aversion: conditional KD of β2 receptors using micro-RNA based interference (miRNAi) reduces pain-induced CPA while their opto-activation on astrocytes promotes aversive memory formation (Iqbal et al., 2023). This is particularly interesting as it has been reported that overactivation of microglia in the SC is involved in pruning inhibitory synapses and promoting pain sensitization (Yousefpour et al., 2023). Given the ACC also exhibits a reduction in GABAergic signaling in neuropathic conditions, NAergic overactivation of microglia could play a similar role in cortical circuits (Blom et al., 2014). There is evidence that DA, by chemical similarity with NE, can also bind to α1 noradrenergic receptors expressed on astrocytes in the ACC and this α1-mediated catecholaminergic pathway may play a role in cognition. (Pittolo et al., 2022).

Due to the excitatory effect of NE on pyramidal excitability, activation of PFC-projecting LC fibers causes aversion and exacerbates pain perception whereas microinjection of an α1 antagonist in the ACC is analgesic (Kaushal et al., 2016; Hirschberg et al., 2017; Koga et al., 2020). In chronic pain states, the LC displays hyperexcitable characteristics and there is anatomical evidence of NE fiber sprouting in the ACC in neuropathic rodents (Cordeiro Matos et al., 2018; Camarena-Delgado et al., 2022). What causes the increase in cortical adrenergic activity is not established yet, but similarly to the situation in the VTA, ineffective MOR activation by endogenous opioids in ACC-projecting LC neurons may be causing a disinhibition in chronic pain states (Guajardo et al., 2017). The pathway leading to an increased NEergic excitation of pyramidal neurons in the ACC in chronic pain conditions could become a therapeutic target. However, due to the analgesic effects of spinal NE, designing a clinical approach that exclusively targets NE signaling in the mPFC remains a challenge.

## Serotonin

**FIGURE 3:**
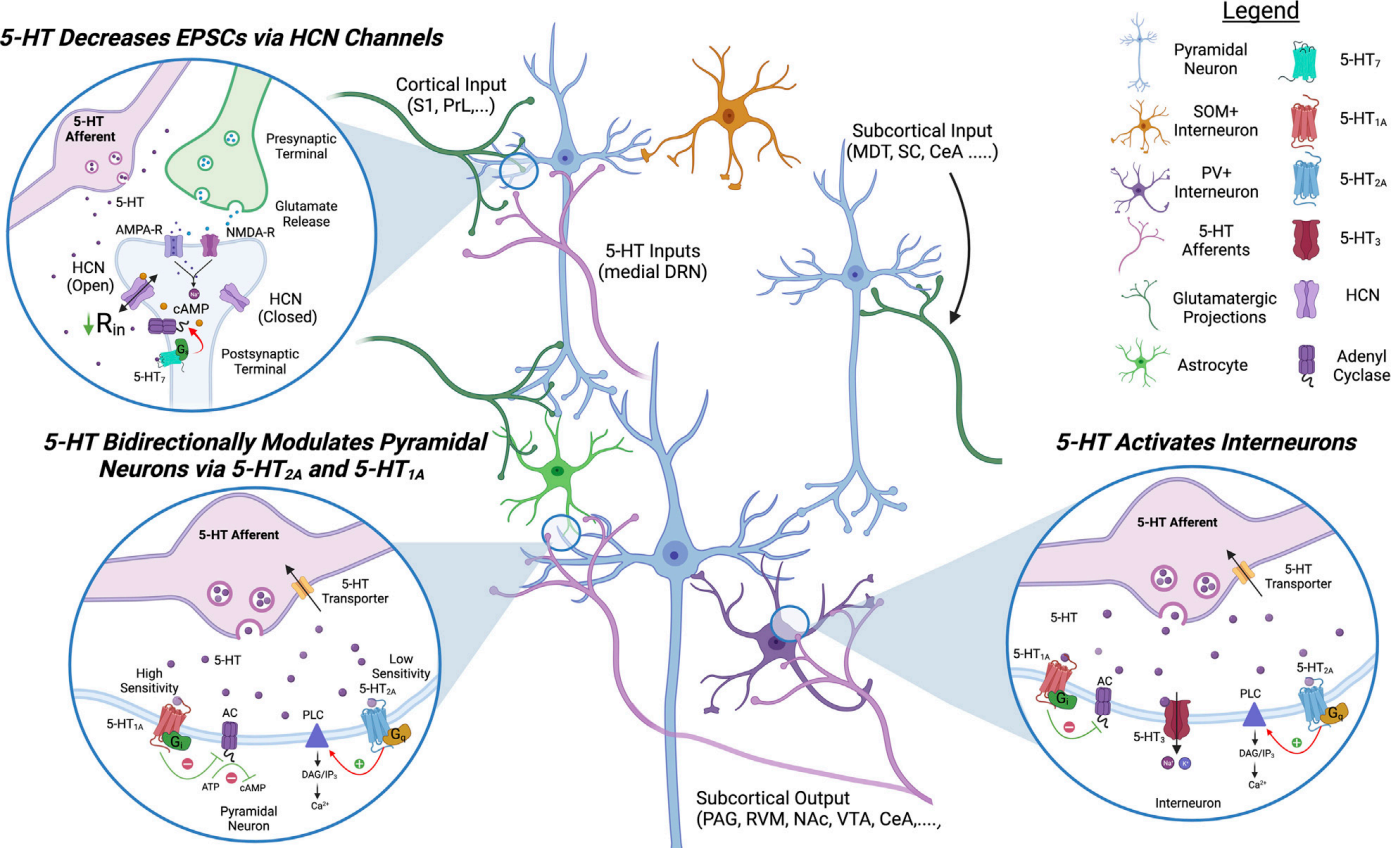
**Proposed Model of Serotonergic Modulation of ACC Circuits**. 5-HT signaling in the ACC inhibits pyramidal neurons and activates inhibitory interneurons. 5-HT_7_ decreases EPSCs on pyramidal neurons by increasing the open channel probability of HCN channels. 5-HT_1_ and 5-HT_2_ bidirectionally modulate pyramidal activity via G_i_- and G_q_-signaling, respectively. 5-HT_2_ and 5-HT_3_ signaling activates inhibitory interneurons. 5-HT, serotonin; HCN, hyperpolarization-activated cyclic nucleotide-gated channels; AC, adenyl cyclase; PLC, phospholipaseC; PAG, periaqueductal gray; S1, somatosensory cortex 1; PrL, prelimbic cortex; MDT, medial dorsal thalamus; SC, spinal cord; CeA, central nucleus of amygdala; RVM, rostral ventromedial medulla; NAc, nucleus accumbens; VTA, ventral tegmental area.

5-HT projections from the medial dorsal raphe nucleus (DRN) to the ACC have also been shown to modulate E/I balance and pain sensitivity. Although DRN projections to the ACC have been identified using retrograde labeling, little is known about their response to both acute pain stimulation and whether they are dysregulated in chronic pain conditions (O’Hearn and Molliver, 1984; Sarter and Markowitsch, 1984; Waselus et al., 2011). Fortunately, due to the established role of 5-HT in psychosis, the impact of 5-HT on ACC circuits is well characterized (Figure 3).

Pyramidal neurons have been shown to express the receptors 5-HT_1A/B_, 5-HT_2A_, 5-HT_7_, and 5-HT_4_; and 5-HT application on acute brain slices is generally inhibitory in synaptically isolated pyramidal neurons (Tanaka and Alan North, 1993; Santana et al., 2004; Santello et al., 2017; Tian et al., 2017). However, selective activation of postsynaptic 5-HT_1A_ or 5-HT_2A_ receptors appears to have opposite effects: agonists of G_i_-coupled 5-HT_1A_ hyperpolarize pyramidal cells, reduce EPSCs and NMDA currents, while selective activation of G_q_-coupled 5-HT_2A_ depolarizes pyramidal cells (Araneda and Andrade, 1991; Tanaka and Alan North, 1993; Avesar and Gulledge, 2012; Tian et al., 2017). As the 5-HT_2A_ receptor has a lower affinity for 5-HT than 5-HT_1A_, an increase in 5-HT release could turn an inhibitory response into an excitatory response. The G_s_-coupled 5-HT_7_ receptor, located on apical dendrites in L5, has also been demonstrated to modulate HCN channels similarly to D1R. Selective 5-HT_7_ agonists inhibit pyramidal neurons via opening of HCN channels and treatment with the 5-HT_7_ agonist LP-211, permeable to the blood-brain barrier, alleviates symptoms of chronic pain in animal models (Santello and Nevian, 2015; Santello et al., 2017). Less is known about the overall effects of G_s_-coupled 5-HT_4_ receptor in the ACC circuits as its activation has mixed effects of pyramidal excitability (Yan, 2002).

The 5-HT_1A_, 5-HT_2A_, and 5-HT_3_ receptors are also expressed on GABAergic interneurons (Zhou and Hablitz, 1999; Yan, 2002; Santana et al., 2004; Puig et al., 2010). Roughly 30% of PV+ interneurons express 5-HT_1A_ or 5-HT_2A_ and the 5-HT_2A_ receptor is expressed on somatostatin (SOM+) interneurons in neighboring cortical regions (Puig et al., 2010; De Filippo et al., 2021). 5-HT_1A_ activation on fast spiking interneurons in the ACC decreases interneuron activity while 5-HT_2A_ activation increases it (Puig et al., 2010). The 5-HT-gated cation channel 5-HT_3_ depolarizes GABAergic interneurons and increases IPSCs on pyramidal neurons (Zhou and Hablitz, 1999; Férézou et al., 2002; Puig et al., 2004). The role of cortical 5-HT on pain responses is less characterized than DA or NE but there is evidence the 5-HT_2A_ and 5-HT_7_ receptors play a role in sensory discrimination and pain sensitization (Gresch et al., 2007; Santello et al., 2017).

At the source of cortical 5-HT projections, it has been reported that the DRN can be inhibited but also activated by noxious stimulation (Sanders et al., 1980; Porro et al., 1991). A study using microdialysis indicates increased 5-HT concentrations in the PFC of neuropathic mice following DRN stimulation (Ito et al., 2013) Modulating GABAergic interneurons in the DRN can bidirectionally modulate nociception: activation of DRN-GABA+ neurons is anti-nociceptive while inhibition of DRN-GABA+ is pro-nociceptive (Xie et al., 2022). Additionally, there is a high degree of crosstalk between the DRN and VTA, likely affecting cortical DA release (Wang et al., 2023). Recent findings indicate that VGluT3+ DRN projections to the VTA play a role in tuning pain perception by modulating DA release in the striatum and that this pathway is inhibited in neuropathic conditions, decreasing glutamatergic drive on DAT+ VTA neurons (Wang et al., 2023). Further research is required to determine how the DRN and its projections to the ACC are affected by the onset of chronic pain.

## Acetylcholine

ACh, originating from the basal forebrain, is another neuromodulator that has a strong impact on mPFC circuitry and is well studied for its role in sustained attention and working memory (Passetti et al., 2000; Howe et al., 2017). The ACC expresses both nicotinic ACh receptors (nAChRs), consisting mostly of the α7, α4, and β2 subunits, and muscarinic receptors (mAChRs), mainly the subtypes M_1_, M_2_, and M_4_ (Albuquerque et al., 2009). Cholinergic projections to the mPFC can be separated into 4 distinct pathways, each innervating different regions of the mPFC with different modulatory effects (Bloem et al., 2014; Oswald et al., 2022). Due to the expression of a wide range of cholinergic receptors and distinct cholinergic inputs, the effect of ACh on ACC circuitry is situationally dependent as it can both inhibit or excite neurons depending on the situation, and whether ACh is release phasically or tonically (Gulledge and Stuart, 2005; Aracri et al., 2010). nAChRs are ACh-gated cation channels expressed on both GABAergic interneurons and pyramidal neurons in the ACC and their activation impacts ACC circuitry differently depending on the cortical layer. In L2/3 nAChR activation is inhibitory to pyramidal neurons due to the expression of nAChRs on GABAergic somata and terminals whereas in L5/6 nAChR activation induces pyramidal firing due to a higher expression on pyramidal neurons (Araci et al., 2010; Poorthuis et al., 2013). Although nAChRs are also expressed on GABAergic interneurons in L5/6, their effect is dependent on glutamatergic transmission and their activation can be either excitatory or inhibitory on interneurons (Vidal and Changeux, 1989; Gulledge and Stuart, 2005; Aracri et al., 2010).

The mAChRs are GPCRs expressed on pyramidal neurons in L2/3/5/6 and are generally excitatory to L5/6 but can induce inhibition in L2/3 due to dual expression on PV+ GABAergic interneurons (Disney and Aoki, 2008; Shirey et al., 2009). Selective M_1_ agonists increase EPSCs in the mPFC L5/6 and can induce persistent firing, the neuronal substrate for working memory (Zhang and Philippe, 2010; Kamiński et al., 2017). Due to this, mAChRs play a major role in modulating sustained memory tasks in rodents and primates (Shirey et al., 2009; Galvin et al., 2020).

In pain, cholinergic projections from the medial septal nucleus (MSN) to the ACC is pronociceptive and stimulation of these cholinergic projections is hyperalgesic in inflammatory models of chronic pain (Jiang et al., 2018). Conversely, chemogenetic inhibition of the MSN to ACC pathway is analgesic and produces conditioned place preference (CPP) in chronic pain (Jiang et al., 2018). Scopolamine, a nonspecific muscarinic antagonist, is commonly used for its anti-depressive effects and microinjection into the ACC produces anti-nociceptive effects, possibly by inhibiting nociception-related attention (Ortega-Legaspi et al., 2003). However, due to M_1_ expression on GABAergic interneurons and the differential effect of ACh depending on physiological state, increased ACh release can be analgesic: microinjection of the M_1_ agonist McN-A-343 into the ACC increases mechanical nociceptive thresholds via promoting GABA release (Koga et al., 2017).

Although it is unknown if chronic pain states disrupt the release of ACh in the ACC, the monoamines DA and NE play an active role in modulating mAChR-induced persistent firing (Zhang et al., 2013; Lançon et al., 2021). Therefore, we can safely infer that dysregulation of their release affects the functionality of cholinergic signaling in the ACC. In the striatum, ACh has also recently been shown to generate action potentials in distal DA axons via activation of nAChRs, promoting the local release of DA (Liu et al., 2022). Therefore, alterations in the release of ACh could be affecting the local release of DA in the ACC through a similar crosstalk, and this merits further investigation.

## Oxytocin

Neuropeptides and other intercellular mediators have also been shown to significantly affect ACC circuitry and are affected by the onset of chronic pain. The neuropeptide oxytocin (OXT) specifically appears to play a key role in modulating ACC outputs as oxytocin microinjection in the ACC increases mechanical withdrawal thresholds (Li Anna et al, 2021). Physiologically, OXT is released in the ACC via projections from the paraventricular nucleus (PVN) in hypothalamus. In agreement with the analgesic effects of OXT microinjection in the ACC, optogenetic stimulation of the PVN to ACC pathway is also anti-nociceptive (Li Xu-Hui et al, 2021). This effect appears mediated by both the modulation of pyramidal neurons and GABAergic interneurons via the G_q_-coupled oxytocin receptor (OXTR) and vasopressin 1A receptor (Schorscher-Petcu et al., 2010). OXT application in acute brain slices significantly decreases E/I ratio by decreasing EPSCs and increasing IPSCs on pyramidal neurons (Li Anna et al, 2021). This is mainly mediated by activation of GABAergic interneurons: oxytocin increases the resting membrane potential and decreases the rheobase of interneurons but not pyramidal cells. In chronic pain, OXT levels in the ACC are unchanged but the OXTR expression is significantly increased (Li Xu-Hui et al, 2021).

## Connecting chronic pain with attentional dysfunction

Chronic pain patients commonly report deficits in working memory, emotional regulation, attention, and anxiety; all of which have been shown to be processed by the ACC (Rudy et al., 1988; Hart et al., 2003; Apkarian et al., 2004; Grégoire et al., 2012; Guerreiro et al., 2022). There is an overwhelming overlap between the cognitive deficits commonly seen in chronic pain patients and the cognitive roles associated with the ACC, hinting that a dysregulated ACC could be a central hub for chronic pain-induced cognitive deficits (Kummer et al., 2020). This link works in the other direction as well, as patients with pathological deficits in attention control and working memory, such as ADHD and PTSD, display a high prevalence of chronic pain (Brennstuhl et al., 2015; Kind and Otis, 2019; Kasahara et al., 2020; Battison et al., 2023). Pain, being one crucial survival signal from an evolutionary standpoint, demands our immediate attention; thus, it is understandable why it is so deeply integrated into our cognitive processes, and why chronic pain can exert such a profound influence on our attention.

The Arnsten group has repeatedly shown that the homeostatic balance of DA and NE in the mPFC is key to tuning attention in primates and changes in the cortical release of these neuromodulators is associated with attention disorders such as ADHD and PTSD (Arnsten, 2000; Vijayraghavan et al., 2007). Since chronic pain also influences the ratio of NE/DA release in the ACC, it may explain why chronic pain patients have a high prevalence of attention deficits and why there is a high comorbidity between attention disorders and chronic pain. Both ADHD and chronic pain states could result in a similar shift in the differential release of NE and DA, explaining the significant overlap in their symptoms. Similarly, depressive and Parkinsonian patients have decreased DAergic signaling and commonly report attention deficits as well as chronic pain (Mehler-Wex et al., 2006; Zhou et al., 2012).

ACC pyramidal neurons are capable of glutamatergic and cholinergic persistent firing, a non-synaptic cellular substrate for working memory, and it has been demonstrated that DA and NE bidirectional control the duration of this persistent activity: DA decreases it while NE increases it (Zhang et al., 2013; Kamiński et al., 2017; Lançon et al., 2021). Therefore, in neuropathic conditions, the disrupted homeostatic ratio in DA/NE release leads to a dysfunction in how persistent firing is initiated and maintained, likely contributing to deficits in sustained attention and working memory.

Furthermore, decreased levels of DA in the ACC, together with increased levels of NE, decreases the rheobase of pyramidal neurons, making them easier to fire ectopically. Chronic pain-induced increase in the likelihood of pyramidal activation with any given stimulus leads to disruptions in what we pay attention to. Recent endoscopic imaging studies conclude that the proportion of nociception-sensitive neurons in the ACC is increased in neuropathic conditions (Li Anna et al, 2021; Acuña et al., 2023). Behaviorally, a disruption in the filtering process required for sensory discrimination might explain why a noxious stimulus specifically receives our attention in normal conditions and why an innocuous stimulus can be perceived as painful in neuropathic conditions (Vander Weele et al., 2018). This filtering mechanism in the ACC could underlie a key cortical mechanism sensitive to modulation by monoamines that can underlie allodynia and spontaneous pain in chronic pain patients. Persistent pain pathologically sensitizes our attention, increasing both pain-related and innocuous attention signals, shifting the homeostatic balance of attention to innocuous somatosensory, auditory, and visual stimuli. In this regard, chronic pain can be considered an attentional disorder.

Given this, the analgesic effects of monoamine signaling in the ACC likely stems from their influence not only on the emotional valence of pain, but also through their role in tuning our pain awareness. This might explain the role of the ACC in modulating the sensory aspect of pain since pain thresholds, commonly assayed via withdrawal and threshold tests such as the von Frey and the Hargreaves assays, are a function of both peripheral and central sensitivity to external stimuli. Schizophrenic patients commonly report pain insensitivity and have increased DAergic and serotonergic signaling in the brain (Dworkin, 1994; Stahl, 2018). As their peripheral nociceptive nerves are functional and they do not exhibit any somatosensory deficits apart from abnormal perception, this pain insensitivity is potentially mediated by the PFC (Chang and Lenzenweger, 2005). This is supported by EEG studies, which show that peripheral nerve stimulation in schizophrenic patients leads to reduced response amplitudes in the dlPFC and ACC, but not in somatosensory cortex (Huang et al., 2010; Daskalakis et al., 2020). Therefore, analgesia induced by microinjection of DA or 5-HT in the ACC is plausibly functioning by decreasing pain awareness rather than pain sensation.

In light of this, current treatments aimed at treating chronic pain can be somewhat effective at decreasing the sensory symptoms, but because they are mainly peripherally acting, they do not alleviate the cognitive deficits (Schiltenwolf et al., 2014). Systemic gabapentin, a commonly prescribed analgesic, leads to even greater cognitive dysfunction in chronic pain patients (Grégoire et al., 2012; Shem et al., 2018). Centrally acting non-selective monoamine reuptake inhibitors show some efficacy at treating the cognitive deficits in chronic pain. The non-selective NE and 5-HT reuptake inhibitor duloxetine, a commonly prescribed analgesic for cancer treatments, alleviates chronic pain-induced cognitive, but not emotional, symptoms (Grégoire et al., 2012; Chu et al., 2015).

## Next steps and conclusion

**FIGURE 4:**
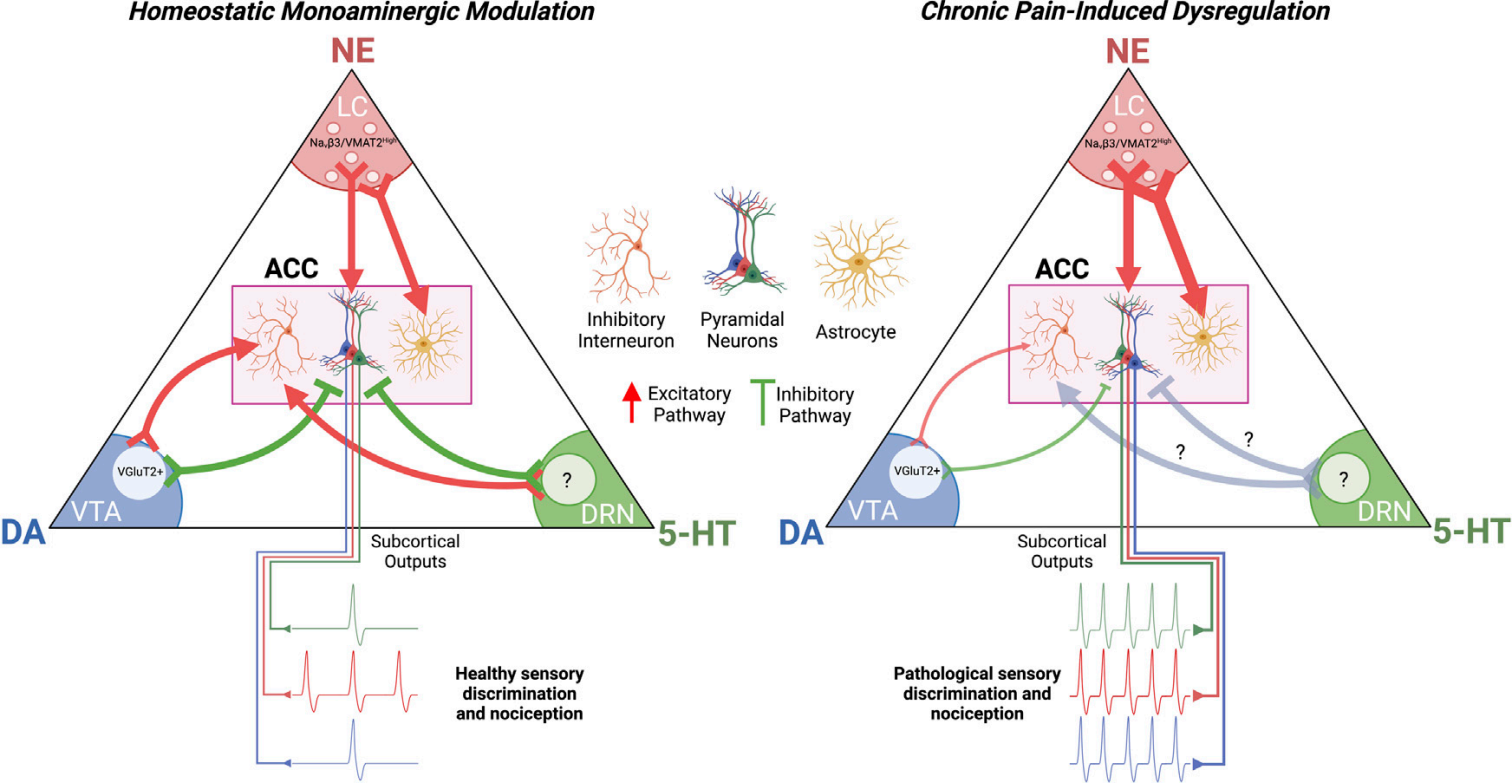
**Chronic Pain-Induced Dysregulation of Cortical Monoamine Signaling**. Chronic pain potentiates NAergic signaling in the ACC while decreasing DAergic signaling. This increases excitatory drive on pyramidal neurons while decreasing excitatory drive on inhibitory interneurons. The effect of chronic pain on 5-HT signaling in the ACC is not established. The resultant effect of dysregulated monoaminergic signaling in the ACC is pathological nociception and a decrease in sensory discrimination. ACC, anterior cingulate cortex; LC, locus coeruleus; VTA, ventral tegmental area; DRN, dorsal raphe nucleus.

Dysregulation of monoamines and other neuromodulators in the ACC in chronic pain as a main contributor to pathological cortical hyperexcitability and chronic pain symptoms is now a widely accepted working hypothesis in the pain research community (Ong et al., 2019; Tan and Kuner, 2021; Kasanetz et al., 2022). The controlled balance of G_s_/G_i_-signaling in specific ACC neurons, as well as in their astrocytic and microglial partners, is key for the healthy integration of innocuous and nociceptive inputs reaching the pain matrix (Figure 4). Disruptions in this balance in one direction leads to hypersensitivity for both nociceptive and innocuous signals while disruptions in the other direction can induce dissociation and pain insensitivity (Dworkin, 1994). Although key neuromodulatory pathways have been identified as instigators of pathological pain signaling, there are still holes in the literature that need to be addressed.

For instance, how chronic pain influences ACh and 5-HT inputs in the ACC has yet to be investigated and could provide insight into their roles in modulating normal and pathological pain perception. In the LC and VTA, it remains unclear what is prompting the changes in their activity and these midbrain regions should be further studied in neuropathic conditions. Additionally, what causes a loss of inhibitory synapses in the neuropathic ACC has yet to be identified. This could be mediated by overactivation of astrocytes and/or microglia but could also be mediated by abnormal corelease of DA with the classical neurotransmitter glutamate. There is precedence for glutamate originating for DAT+ projections forming asymmetrical synapses on PV+ interneurons and dopamine can form hub synapses with both glutamatergic and GABAergic synapses (Qi et al., 2016; Paget-Blanc et al., 2022). As the D1R was found to be expressed on the presynaptic GABA terminals innervating pyramidal cells, it is possible DA is reinforcing these synapses as well as activating PV+ interneurons with glutamate (Muly et al., 1998). The role of KORs in modulating DA release in the ACC also deserves serious investigation.

Moreover, it is intriguing, and apparently contrary to expectation, that G_s_ activation via DA/NE/5-HT signaling on pyramidal neurons in the ACC results in inhibition, whereas G_i_ activation leads to excitation. This phenomenon seems to arise from the modulation of HCN channels through cAMP and their consequent impact on input resistance. Nevertheless, further research is required to determine all the downstream effects of PKA activation.

Central monoaminergic pathways also exhibit a high degree of sexual dimorphism and should be studied further in this regard. For example, the female hormone estradiol has a robust excitatory effect on VTA neurons and could explain why L-DOPA is more successful in females than males at decreasing symptoms of lower back pain (Yoest et al., 2018; Reckziegel et al., 2019). The LC also exhibits prominent sex differences: MOR activation on LC neurons causes a dramatic reduction in NE release in males, but not females (Bangasser et al., 2011; Guajardo et al., 2017).

In addition, this review focuses on the ACC but more attention should be given to how monoamines affect other cortical regions of the pain matrix dysregulated in chronic pain states. The neighboring prelimbic (PrL) cortex is particularly intriguing due to the opposite effect chronic pain induces relative to the ACC: PrL neurons are hypoactive in neuropathic conditions and their activation is analgesic (Wang et al., 2015). Since the PrL cortex also receives dense monoaminergic inputs, it would be interesting to understand how alterations in the release of monoamines induces hypoactivity rather than hyperexcitability. Additionally, the insular cortex appears to play a larger role in modulating pain perception in primates than in rodents. It deserves more investigation as it has also been identified as one of the most active brain regions in neuropathic patients (Jensen et al., 2016; Lu et al., 2016; Ferraro et al., 2021; Tan and Kuner, 2021).

Lastly, although ACC activity is positively correlated with pain sensitization, this is not always the case. Recent reports indicate that gabapentin and nitrous oxide can in fact induce an increase in ACC activity alongside analgesia (Acuña et al., 2023; Weinrich et al., 2023). Given this, the role of the ACC in modulating attention and pain perception is more nuanced than it appears and it may be possible to reinstate proper somatosensory filtering by increasing the activity of specific ACC circuits.

Although further studies are warranted to determine the impact of chronic pain on supraspinal regions involved in the processing of nociceptive information, tremendous progress has been made in the past decade. Our current model remains incomplete however and finding a treatment that selectively corrects cortical monoaminergic function in chronic pain might be key to the development of novel therapeutics.
